# Esophageal rupture through extreme sadomasochistic practice

**DOI:** 10.1007/s00414-023-02972-9

**Published:** 2023-02-18

**Authors:** Sarah C. Koelzer, Lena M. Bunzel, Franziska Holz, Christoph G. Birngruber, Marcel A. Verhoff

**Affiliations:** Institute of Legal Medicine, University Hospital of Frankfurt, Goethe University, Kennedyallee 104, D-60596 Frankfurt/Main, Germany

**Keywords:** Sadomasochism, BDSM, Mutilation, Sexual assault, Esophageal rupture

## Abstract

We report the case of a woman in her thirties who suffered an esophageal rupture while participating in extreme sadomasochistic practices. After herself seeking help in a hospital for complaints alleged to be from a fall, she was initially diagnosed with several broken ribs and a pneumothorax. The cause of the pneumothorax was later discovered to be an esophageal rupture. When confronted with this atypical injury for a fall, the woman admitted to have accidentally swallowed an inflatable gag, which her partner had afterwards inflated. In addition to the esophageal rupture, the patient also had numerous other externally visible injuries of various ages, reportedly also from sadomasochistic acts. Although an in-depth police investigation was conducted and a “slave contract” was found, the woman’s consent to the extreme sexual practices performed by her life partner could not be substantiated conclusively. The man was convicted for intentional infliction of serious as well as dangerous bodily injury and sentenced to a long term in prison.

## Introduction

Sexual activities, whether autoerotic, interpersonal consensual, or against a person’s will, can lead to undesirable outcomes, injuries, or criminal investigations. Subsequently, a forensic medical examination may be necessary to obtain objective findings that allow reconstructive statements or serve to objectify statements about courses of events and actions. Such examinations occasionally occur in the everyday medicolegal work and include, for example, autoerotic accidents with a lethal outcome [1–3], clinical forensic medical examinations after a reported rape [4, 5], or the insertion of objects in body orifices [1, 6–9]. The medicolegal assessment of physical injuries specifically due to sadomasochistic practices is very rare. Only single cases are reported in the medicolegal literature. Fanton et al., for example, describe the case of a 34-year-old man who sustained anal injuries (in part, through insertion of a metal dildo) while engaging in sadomasochistic activities [10]. In another instance, Tanabe et al. report a case in which lesions on a 49-year-old man’s thighs and groins, which initially had been diagnosed as pyoderma gangrenosum, were later found to be burn injuries inflicted during sadomasochistic acts [11]. In a further reported case, a 35-year-old man presented at a venereology department because of wound healing problems of perianal burn wounds inflicted by lit cigarettes during sadomasochistic activities [12].

We would like to add to this body of literature by reporting on a case in which a rare, life-threatening injury and an extreme extent of further injuries were inflicted during sadomasochistic acts.

## Case history

A woman in her thirties presented in the emergency department of a hospital with symptoms of radial nerve palsy on her right side. She reported to have fallen in the shower a few days earlier. The attending doctor noticed that she had tried to cover up a bruise on the outer edge of her left eye socket with make-up. At the time he did not question the injury mechanism. He conducted a clinical neurology examination, noted paresis of the right radial nerve, and referred the woman to a neurologist.

Five days later, the woman again presented in the same emergency department, this time with extreme breathing problems and in extremely poor general condition. Chest radiography showed a complete right-sided pneumothorax, opacity over the right hemithorax that suggested pleural effusion, and several recent, as well as older, rib fractures. The woman continued to attribute her injuries to falling in the shower. Two thoracic drainages that were subsequently performed produced such large quantities of heterogeneous exudate that a solely pulmonary cause for the pleural effusion seemed improbable. After a contrast-swallow test, a 10-cm-long tear in the distal esophagus could be identified on the esophagogram. Upon being confronted with this finding, the woman admitted to have “accidentally” swallowed an approximately 10-cm-long inflatable gag attached to a 40-cm-long inflation tube. The gag had still been deflated when she had swallowed it. She reported the gag had actually been intended for insertion into the trachea. The woman was in critical condition and in an induced coma for several days following surgery to recover the esophageal rupture. The attending doctors discovered that their patient had “multiple hematomas on her entire body” and that “both nipples were missing.” A gynecological consultant was, therefore, called in to examine the woman. Subsequently, the police were informed and an investigation was launched. Questioning of the woman’s partner (who was a few years older) revealed that he and the patient had “extreme sexual preferences” and that the woman, in particular, had had “extreme sexual proclivities” that he had only “accommodated.” As “proof of consent,” he presented the police officers with a “slave contract,” in which the woman declared herself to be the man’s property, and in which she gave her unconditional consent to all activities, including torture. The inflatable gag was found in a search of the house, along with numerous other items used in sadomasochistic acts. Furthermore, a large wooden St. Andrew’s cross with fetters was found, as well as an abundance of self-recorded pornographic video footage depicting the woman and her partner.

**Fig. 1:**
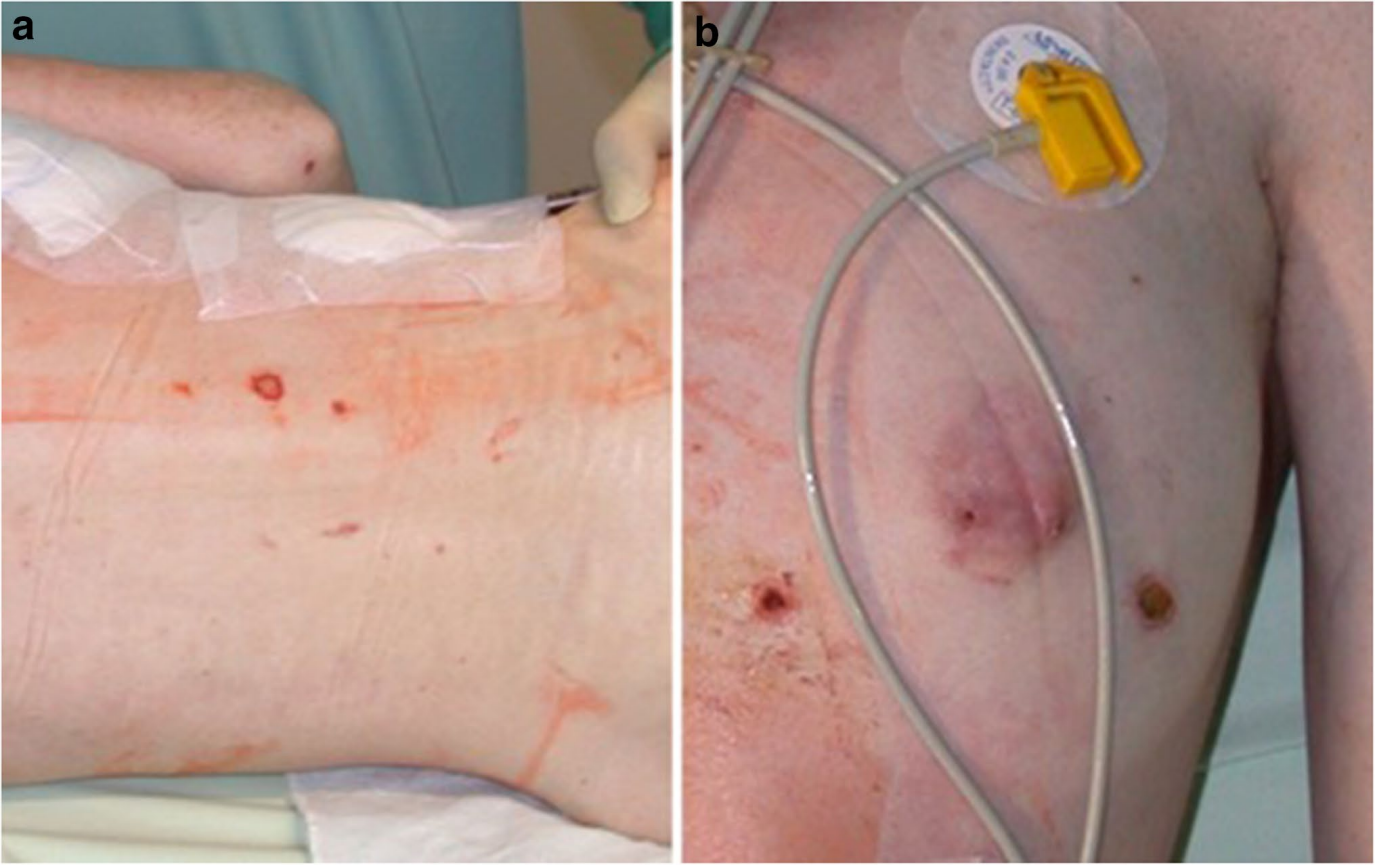
**a**, **b** Skin on chest and lower back (left image) and skin on left breast (right image), with partially missing mammilla, scarred areola, and burns, most likely from cigarettes

**Fig. 2:**
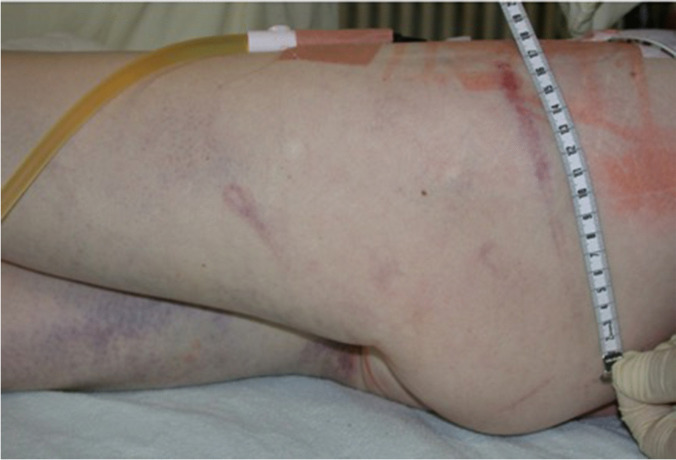
Discolorations, in part like tram-line bruises on thighs and buttocks

The investigating authority subsequently ordered medicolegal examination of the woman. The findings were as follows: The woman was undernourished (marasmus). Both of her nipples were missing, with only residual, scarred areolas. There were numerous lesions on both of her breasts, as well as on her back, which were interpreted as burn injuries caused by lit cigarettes. In addition, there were both older and somewhat more recent wounds caused by nipple piercings (Fig. 1). The woman also had numerous infected wounds on the labia majora and minora that reportedly stemmed from piercings and the use of genital padlocks. There were hematomas on the inner sides of both her thighs. The various shades of discoloration suggested that the hematomas had developed at various times. There were also patterned hematomas in the gluteal region, which were interpreted as tram-line bruises, being caused by caning and whipping (Fig. 2). Furthermore, there was an amateurishly executed tattoo on the woman’s mons pubis, reading “Property of Mr. […]” (anonymized), in addition to both recent and older vaginal and anal lacerations.

In the course of further investigations, the woman’s partner was reported to have burned away her nipples with glowing cigarettes several months earlier. The right-sided radial nerve palsy had been the result of a sadomasochistic act during which the woman reported to have been completely wrapped in cling film by her partner and then suspended in a doorframe by her hands and feet, over hours.

On the basis of the investigation and examination results, the woman’s partner was indicted for serious bodily injury and attempted homicide and the case went to trial. In this context, it also emerged that the woman’s partner had with increasing intensity and frequency inflicted numerous injuries on her over a period of several months, often while she was strapped to a large wooden St. Andrew’s cross. Prior to the offense-related incident, this behavior had escalated to point that the woman had been gagged, with her arms tied behind her back, and suspended by her breasts during sexual activities. This act was accomplished by horizontally piercing steel skewers through her breasts. Through attachments on the left- and right-sided ends of the skewers, the woman could be pulled up and freely suspended.

In a forensic psychiatric assessment, the accused man was found to have a sexual deviation in the sense of a sexual sadistic disorder on the basis of the diagnostic criteria listed in the (then applicable) DSM-5. This sexual deviation had, however, at no time attained the quality of a “compulsive sexual disorder,” and the man had, at all times, been in control of his impulses. The woman, in contrast, was found to have a dependent personality disorder, also based on the diagnostic criteria listed in the DSM-5.

According to the available court verdict, the woman’s partner was sentenced to a long term in prison without parole for the intentional infliction of serious bodily injury in one count and dangerous bodily injury in another count, because he had a history of previous convictions for numerous offenses (e.g., document fraud, negligent behavior in traffic, hit and run, intimidation, and possession of illegal pornographic materials) and was not found to have diminished responsibility. He committed suicide in the penitentiary one and a half years into his prison term.

Despite the found “slave contract,” a full investigation, and the insights gained in the main hearing of the trail, the question of the woman’s consent could, lastly, not be sufficiently clarified.

## Discussion

In the reported case, a woman in her thirties had sustained an esophageal rupture after accidentally swallowing an inflatable gag, which her partner had later inflated during sadomasochistic activities. The woman had almost died in the aftermath of this act. Despite an in-depth investigation, the woman’s consent to these extreme sex practices could not be conclusively proven.

Historically, the terms “sadism” and “masochism” have been categorized for the first time by Richard von Krafft-Ebing in 1886, referring to the French aristocratic Marquis de Sade and the Austrian writer Leopold von Sacher-Masoch. Krafft-Ebing described sexual deviations and perversions in one of the first texts about sexual pathology, the “Psychopathia sexualis” [13]. The term “sadomasochism,” accrued later on, may has resulted by the widespread tendency to live out such preferences with a counterpart, which is slightly replaced by the term of BDSM, subsuming common manifestations such as shackles and discipline (“bondage” and “discipline”), dominance and submission (“dominance” and “submission”), and sadism and masochism in the sense of pain, suffering, and humiliation (“sadism” and “masochism”) [14]. Anyway, the historical definitions of sadism and masochism or sadomasochism, respectively, represented the basis for the diagnostic classification systems ICD and DSM for a long time, in which sadomasochism was classified as a sexual paraphilia, not distinguishing whether these sexual practices were carried out consensual or non-consensual, until the ICD-10 and DSM-IV [13]. However, in the present BDSM subculture, great importance is attached to mutual agreement [13]. This key feature is now to be found in the new ICD-11, valid from January 2022, in which the ICD-10 diagnosis “sadomasochism” (F65.5) [15] is now termed as “coercive sexual sadism disorder” (6D33) [16]. Mokros et al. [14] pointed out three eminent alterations in contrast to the ICD-10 definition, namely the departure from the previous merging of sadism and masochism (sadomasochism) into one single disorder category, the focus on non-consensual manifestations (and thus the distinction from BDSM), and the non-consideration of an undisturbed variant, like in the DSM-5 with the paraphilia (opposite to the paraphilic disorder) is the case.

It can be stated that BDSM is growing in popularity in society, shown by large-scaled studies, by Richters et al. [17], for example. It was conducted on a representative sample of 19,307 respondents from the Australian population, which revealed that 2.2% of the interviewed men and 1.3% of the women reported that they had engaged in sexual activities involving bondage and discipline, sadomasochistic acts, or dominance and submission over the past year. A Belgian study by Holvoet et al. [18] yielded an even higher prevalence of interest in BDSM-related activities in contrast to that by Richters et al., determining that 46.8% of the total sample by the general population had ever performed at least one BDSM-related activity and an additional 22% indicated having (had) fantasies about it. Furthermore, 12.5% of the total population indicated performing at least one BDSM-related activity on a regular basis.

The attempt to distinguish consensual BDSM acts from sexual assault solely on the basis of morphological injuries can be challenging. Song and Fernandes [5], for example, note in their review that it is often impossible to deduce from the morphology of anogenital injuries whether a sexual act was consensual or not, as no difference is found in the frequency, morphology, or location of these injuries between consensual and non-consensual sexual acts. The authors of the review do, however, point out that there were great discrepancies in the available data in the reviewed studies and the methods were also not uniform. Notwithstanding this limitation, Song and Fernandes’ conclusion is also supported by Bunzel et al.’s study, in which 74 non-natural deaths which had occurred in the context of sexual activity were assessed. The authors of that study found that the frequency of anogenital injuries was approximately the same for autoerotic (27%), consensual (26%), and non-consensual (24%) sexual acts. The comparatively small number of cases in each of these groups must, however, be considered a limitation of that study [19]. Sexual practices, including autoerotic ones, that are known to be associated with a risk for life-threatening or fatal injuries encompass those in which objects such as dildos, bottles, or broom sticks are inserted vaginally or anally. This applies equally to so-called fisting or handballing practices, in which a fist (and sometimes also a part of the lower arm) is inserted into the vagina or anus. Practices such as these can cause serious intra-abdominal injuries, such as intestinal perforation or hemorrhage. In their review, Cappelletti et al. provide an overview of cases of “fisting” or “handballing” injuries (*n* = 47) described in the literature and report that internal injuries had been observed in the totality of cases. The observed injuries had, in particular, been lacerations of the rectosigmoid colon, which, in 8 cases, had been fatal [20].

As the case we report demonstrates, BDSM practices should also be kept in mind as possible causes of injury in the forensic interpretation of atypical or initially inexplicable injuries (not only in the anogenital region!) — especially in light of the high prevalence figures to be found in the literature for sadomasochistic fantasies and practices. Not least of all due to the sheer boundless variety of human sexual preferences and associated practices, the interpretation of seemingly inexplicable injuries is an important forensic task, which not infrequently requires a commensurate degree of imagination on part of the medicolegal expert.

## Data Availability

Not applicable.
